# Ganser syndrome and lesion in the temporoparietal region

**DOI:** 10.4103/0019-5545.31605

**Published:** 2006

**Authors:** M. Anupama, K. Nagaraja Rao, S. Dhananjaya

**Affiliations:** * Lecturer Department of Psychiatry, J.J.M. Medical College, Davangere 577004; ** Professor Department of Psychiatry, J.J.M. Medical College, Davangere 577004; *** Postgraduate student Department of Psychiatry, J.J.M. Medical College, Davangere 577004

**Keywords:** Ganser syndrome, dissociation, left temporoparietal bleed

## Abstract

Ganser syndrome is a rare dissociative disorder. It has been reported in association with various functional psychiatric disorders and organic states, most often in patients with head injury and stroke, especially those involving the frontal lobes. The present case of Ganser syndrome had features of hysterical dissociation but was found to have haemorrhage in the temporoparietal region of the dominant hemisphere. The complexities of Ganser syndrome in the presence of an organic lesion with an overwhelming emotional component are discussed.

## INTRODUCTION

Ganser syndrome was first described in 1897 by Sigbert Ganser in 4 prisoners. Initially, it was believed to be rare, occurring mainly in forensic settings. Hence, it was referred to as prison psychosis. Later, such cases were reported more frequently in non-forensic settings.^1^ The syndrome has found a place in both the ICD-10 and DSM-IV, despite controversy about its existence and distinctiveness. This disorder was previously classified as a factitious disorder; currently, it is classified under ‘dissociative disorder not otherwise specified’.

The core clinical features of this syndrome are approximate answers, clouding of consciousness, somatic conversion symptoms and hallucinations. However, all the core symptoms are not needed for diagnosis.^2^ Ganser himself had noted impairment of grasp, attention, concentration, anxiety and perplexity as additional features.^2^ There is a report of this syndrome with the symptom of prosopagnosia as a hysterical feature.^3^ Based on the clinical features it has been variously named as nonsense syndrome, approximate answer syndrome (*vorbeireden* [to pass by]), pseudodementia and balderdash syndrome.

Ganser had considered it as a form of transitory hysterical twilight state with amnesia for the episode on recovery. Psychopathologically, it is believed to provide an escape from an inescapable, intolerable and confining situation.^2^ However, symptoms of Ganser syndrome have been described in schizophrenia,^2^,^4^ affective disorder^2^,^5^,^6^ and organic states.^7^ Ganser syndrome in organic states has mainly been reported in patients with head injury and stroke, mostly those involving the frontal lobes.^7^–^10^ It has also been reported in alcoholism with Korsakoff psychosis,^1^ neurosyphilis,^4^ dementia^11^ and AIDS.^12^ The present case of Ganser syndrome had features of hysterical dissociation but was found to have intracerebral haemorrhage in the temporoparietal region of the dominant hemisphere.

## THE CASE

A 34-year-old male farmer was taken to a general practitioner with the complaints of headache, giddiness, vomiting and irrelevant talk of 1 day's duration. He was managed symptomatically. On the second day, there was no giddiness and vomiting but complaints of headache and irrelevant talk persisted. Hence, he was referred to a physician in a general hospital, who found no obvious abnormalities on general and systemic examination, and referred the patient to a psychiatrist.

Psychiatric evaluation revealed that he was the eldest of the 4 siblings with 2 sisters and a brother. He was married for the past 2 years and had a daughter. Apart from being a farmer he was also an insurance agent. He was the breadwinner and decision-maker in the family. He had incurred loans of about Rs 300,000 to meet the marriage expenses of his sisters. Due to crop failure and increased family expenses he could not liquidate the loans. One month prior to the present illness he had sold part of his land at the suggestion of his parents to lessen his financial burden. He was unwilling to part with the land but felt helpless and wanted to get out of the trap of the huge loans, which carried high interest rates. Since the time he sold his land, he complained of sadness, disturbed sleep and decreased interest in life. In the ward, at times, he complained of inability to see, though there were no behavioural concomitants of blindness. His past history revealed that he had had one episode of transient, self-limiting depression following failure in the standard XII examination.

On Mental State Examination (MSE) he gave approximate and absurd answers. He said that a hen has 4 legs, a car has 2 wheels, a chair has 2 legs and that there were 365 months in a year. He said the month was 8 instead of August, and the year was 24 instead of 2004. When asked what he was sitting on he replied ‘box’ instead of chair. These approximate answers were interspersed with correct answers such as a cow has 4 legs. He said the colour of a red pen was white; however, he was able to name other colours and other red objects correctly. Some of his answers had a paraphasic quality. He pronounced the names of the cities Davangere and Channageri as Davangevi and Channagevi. He identified a pen as a pencil. He said the name of the state was Kannada instead of Karnataka. When this answer was followed by a question asking him to name the country he answered ‘Karnataka’. This answer could be an example of perseveration. His attention, though arousable, was ill sustained. He was oriented to time and place but had difficulty in identifying persons. He identified his younger brother as his older brother and his father-in-law as maternal uncle, though he could tell their names correctly. He identified his mother correctly but could not tell her name. While answering questions, at times he looked perplexed and mildly confused. He reported that he knew the answers but had difficulty in responding as the answers did not come quickly to his mind. On Mini Mental State Examination (MMSE) he scored 10. His memory quotient was 63 on the Wechsler Memory Scale. Though low scores on these tests are supposed to be indicative of gross organic pathology, his clinical assessment and ward behaviour did not support such a conclusion.

The patient was treated with antidepressants and verbal suggestion. As he did not improve he was given mild faradic stimulation as a means of suggestion after obtaining informed consent from both the patient and his parents, keeping in mind the ethical issues involved. He reported significant improvement in headache and was able to talk correctly following suggestion. He voluntarily requested more frequent electrical stimulation. He was also reassured that his decision to sell his land was correct. The patient improved gradually, and his answering pattern was not approximate any more. However, his paraphasic responses and naming difficulties persisted. As there was no progressive and consistent improvement in his symptoms a CT scan was done, which showed evidence of intracerebral haemorrhage in the left temporoparietal area.

## DISCUSSION

Whether Ganser syndrome is primarily a hysterical or an organic phenomenon has been debated in the literature. However, there have been cases with both hysterical and organic elements.^13^ In a case of Ganser syndrome with AIDS it was opined that Ganser syndrome was not due to the direct effects of HIV infection, and psychological factors were of primary importance.^11^ In the present case both hysterical and organic elements were present.

The patient's absurd, approximate and inconsistent answers to simple questions despite his being cooperative led to the diagnosis of Ganser syndrome. The presence of somatic symptoms that waxed and waned, transitory blindness arising in an emotionally charged, helpless situation, suggestibility and perplexity led to the consideration of Ganser syndrome as a form of hysterical dissociation. Diagnoses of malingering and factitious disorder were not considered as the symptoms had a genuine quality, the patient had no overt intention of giving up his responsibilities and there was no obvious gain.

A past history of depression, symptoms of sleeplessness, brooding, lack of interest in work, sadness of mood at onset and the presence of absurd and approximate answers warranted a diagnostic consideration of depressive pseudodementia. This diagnosis was less likely in the absence of objective evidence of sadness and the presence of well-preserved daily activities. Mild depressive symptoms were a reaction to stress.

Organic illness was considered because his symptoms started with headache, transitory giddiness and vomiting. In addition, his cooperative demeanour, the quality of nominal aphasia, paraphasia, perseveration and the perplexity of some of his answers, and ill sustained attention pointed to an organic cause. A CT scan revealed an area of haemorrhage measuring 2.0 × 1.5 cm in the left temporoparietal region.

There have been case reports of Ganser syndrome with an organic aetiology. Most of the reported organic cases have a history of closed head injury, including a few of Ganser's original cases.^8^,^9^ A history of head injury may complicate the issue as such cases may involve monetary compensation. There have been sporadic cases of cerebrovascular accident involving the frontal lobe presenting with symptoms of Ganser syndrome.^10^,^13^ A temporoparietal lesion with Ganser syndrome as described in this case has not been reported in the literature.

In the present case it may be possible that a dissociative mechanism triggered by emotional disturbance contributed to some of the symptoms. In addition, paraphasia, perseveration, perplexity, nominal aphasia, misidentification of familiar persons and ill sustained attention may have been due to the brain lesion. A lesion in the region of the temporoparieto-occipital crossroads is known to give rise to failure to associate and integrate discrete elements into a meaningful whole, giving rise to complex perceptive and cognitive disturbances. Such patients can understand and remember a problem but are unable to carry out the necessary operations to solve it. They may have calculation difficulties as they see numbers in quasispatial relations rather than proper spatial relations such as place value. They may also have communication errors due to disturbance in abstract logical relations of syntax where they may fail to arrange the sequencing of words properly, giving rise to semantic aphasia.^14^ In the present case, we did not find all these characteristic features except that there was place value disturbance in the form of 24 instead of 2004 and paraphasia. The paraphasia in this case involved both the structural and functional aspects of language. The disturbance in the structural aspect was in the form of phonemic (substitution of syllable, e.g. Channagevi, Davangevi) and semantic (substitution of a word, e.g. Kannada, pencil) paraphasia. There was no evidence of neological (substitution of a word by nonsense word) paraphasia. The disturbance in the functional aspect was in the form of categorical (substitution of word with another word of same class, e.g. box for chair) and associative (substitution of a word with another word of the general class, e.g. white for red) paraphasia. There was no evidence of asemantic (substitution of a word with another word having no meaningful relationship to the correct word) paraphasia.

Ouyang *et al.*^10^ have postulated that emotional stress may induce corticolimbic release of glutamate. They further suggest that this hyperglutaminergic transmission in the frontal lobe may lead to dissociative symptoms in cases such as Ganser syndrome, PTSD, stroke, etc. Therefore, coexistence of hysterical dissociation and an organic lesion is patho-physiologically understandable. Such cases call for a collaborative approach to management, especially when there is an associated emotional component.
